# A Multidimensional Analysis of AI Literacy Determinants: External Resources, Digital Skills, and Psychological Profile Among University Students

**DOI:** 10.3390/bs16081448

**Published:** 2026-08-21

**Authors:** Tak Sang Chow, Ken To, Bess Yin Hung Lam, Kung Wong Lau

**Affiliations:** 1 Department of Counselling and Psychology, Hong Kong Shue Yan University, Hong Kong, China; yhlam@hksyu.edu; 2 Research Centre for Digital Education and Blended Intelligence, Hong Kong Shue Yan University, Hong Kong, China; hto@hksyu.edu; 3 Department of Applied Data Science, Hong Kong Shue Yan University, Hong Kong, China; laukw@hksyu.edu

**Keywords:** artificial intelligence, AI literacy, resources, teacher support, digital competence, personal innovativeness, technology anxiety, growth mindset in technology, higher education, Hong Kong, social cognitive theory

## Abstract

External resources, digital competence, and psychological characteristics have each been linked to Artificial Intelligence (AI) literacy, but almost always in isolation, so the unique contribution of any one domain remains unknown. Grounded in social cognitive theory, this study models all three domains jointly. Data were collected from 303 undergraduates at a liberal arts university in Hong Kong and analysed using hierarchical multiple regression. Perceived resources and perceived teacher support explained 29% of the variance in AI literacy; prior digital competence added a further 3%; and personal innovativeness, technology anxiety, and growth mindset in technology added a further 6%, with all three being significant in the final model (total *R*^2^ = 0.39). The external predictors remained significant throughout but attenuated substantially, indicating that institutional provision is necessary but not sufficient. Technology anxiety, which correlated negatively with AI literacy at the zero-order level, emerged as a positive predictor once other determinants were controlled. Analyses of the five AI literacy subdimensions localised this effect to critical evaluation and ethical competence and found no association with the three performance-oriented dimensions, suggesting that anxiety operates through heightened vigilance rather than enhanced operational skill. These findings, which no single-domain design could have produced, indicate that fostering AI literacy requires attention to students’ psychological readiness and foundational digital skills alongside the provision of infrastructure.

## 1. Introduction

### 1.1. The Search for Determinants of AI Literacy

Since the launch of ChatGPT in November 2022, generative artificial intelligence (AI) has profoundly transformed everyday life, becoming integral to personal, professional, and educational activities (Capraro et al., 2024). Within higher education, the advancement and availability of generative AI technology have been shown to promote the adoption of generative AI (X. Zhang et al., 2025), and can enhance the quality of AI outputs (Bećirović et al., 2025). Nevertheless, misuse of generative AI raises concerns not only about academic integrity but also about its potential to hinder learning processes. For example, by reducing students’ critical thinking and problem-solving engagement when overreliance on AI-generated content replaces active cognitive effort (Cotton et al., 2024), and by fostering surface-level understanding rather than deep learning when AI tools are used unreflectively (Zhai, 2023). The extent to which generative AI use benefits or undermines learning outcomes largely depends on users’ level of AI literacy (Bećirović et al., 2025; Ng et al., 2021), underscoring the importance of understanding the factors that contribute to individual differences in these competencies.

AI literacy, understood as a “set of competencies that enables individuals to critically evaluate AI technologies; communicate and collaborate effectively with AI; and use AI as a tool online, at home, and in the workplace” (Long & Magerko, 2020, p. 2), is an emerging skill set necessitated by the rapid advancement of artificial intelligence. While researchers continue to debate the specific dimensions of AI literacy, there is a growing consensus that it prioritizes functional understanding over the mastery of technical programming or machine learning algorithms (Laupichler et al., 2022). For instance, Lee and Park (2024) proposed a framework comprising technical proficiency, critical evaluation, communication proficiency, creative application, and ethical competence. Currently, the prevailing approach to fostering AI literacy in the education system is through direct instruction within formal curricula, particularly in higher education, and provision of resources (S. Zhang et al., 2025). However, teachers face significant challenges in addressing the diverse learning needs of students (Ng et al., 2023; Su et al., 2022). Furthermore, a persistent digital divide has been observed even among university populations (C. Wang et al., 2025). Consequently, identifying the factors that drive individual differences in AI literacy is essential for developing inclusive and effective pedagogical strategies.

Individual differences in AI literacy are particularly salient in higher education, where self-directed learning is emphasized. Understanding the factors that contribute to such disparities is crucial for promoting equitable and responsible AI engagement. As indicated by the body of literature, the development of AI literacy is influenced by a combination of external, experiential, and psychological factors. External resources, including institutional infrastructure, AI training programs, and teacher support, can significantly facilitate or constrain AI literacy, often reinforcing inequities associated with socioeconomic or institutional differences (Almatrafi et al., 2024). Prior digital competence, acquired through previous technological exposure, further shapes individuals’ ability to navigate and effectively utilize AI systems (Chee et al., 2025; Riina et al., 2022). Psychological traits and skills, including cognitive absorption and emotional intelligence, play a critical role in promoting engagement with generative AI and supporting its integration into self-directed learning (Celik, 2023; Fu, 2025). Together, these dimensions not only contribute to positive outcomes, such as enhanced human–machine collaboration, but also help mitigate potential risks, including overreliance on AI and ethical lapses (Cotton et al., 2024). Understanding the interplay among these factors is therefore essential for explaining individual differences in AI literacy and for informing interventions that foster equitable and responsible engagement with generative AI in higher education context. Nevertheless, the existing evidence base supports these claims less securely than its volume suggests, and four limitations warrant attention. First, the great majority of studies estimate the effect of a single determinant, or of several determinants drawn from the same domain, so correlated influences originating in the other domains are left unmodelled. Second, much of the psychological evidence derives from teacher, researcher, and professional samples (Dringó-Horváth et al., 2025; Fu, 2025; Seker et al., 2025) rather than from undergraduates, despite higher education being the setting in which self-directed AI learning is most consequential. Third, studies rarely specify which dimension of AI literacy a given determinant affects, treating the construct as undifferentiated even where multidimensional instruments have been administered. Each of these limitations is addressed in the design of the present study.

This study investigates how external resources, prior digital competence, and psychological profiles collectively shape AI literacy among undergraduates in a liberal arts university in Hong Kong. Specifically, external resources are conceptualized as perceived resources and perceived teacher support; prior digital competence reflects individuals’ prior technological exposure; and psychological profiles include personal innovativeness (trait), technology anxiety (emotion), and growth mindset in technology (belief). Drawing on Celik’s (2023) multidimensional framework, which emphasizes that skill development arises from the interplay of psychological, environmental, and behavioral factors, this study adopts an integrative perspective. While prior research has identified individual differences in AI literacy and explored selected factors in isolation (e.g., Shen & Cui, 2024; K. Wang et al., 2025), few studies have examined how external resources, digital competence, and psychological profiles jointly influence AI literacy. Addressing this gap, the present study integrates these dimensions to provide a comprehensive understanding of the determinants of AI literacy. This holistic approach offers empirical insights that can inform targeted interventions to enhance AI literacy.

Two commitments therefore guide this study, and it is worth stating at the outset why they are complementary rather than in tension. The first is integrative. External resources, prior digital competence, and psychological profile must all be represented if AI literacy is to be accounted for adequately. The second concerns incremental contribution, and gives particular attention to what psychological factors add once the other two domains are taken into account. These may appear to pull in opposite directions, since the first treats the three domains as jointly necessary while the second appears to single one of them out. The appearance dissolves once it is recognised that incremental value is not an intrinsic property of a predictor but a relational one. To say that psychological factors contribute incrementally is to say something about their relation to external resources and prior digital competence, and the statement has no content in the absence of those comparators. A single-domain study can establish that psychological factors are associated with AI literacy; only an integrated model can establish that they add anything to what is already known. The second commitment thus depends upon the first rather than competing with it, in the way that a statement about relative height depends on there being more than one object in view.

### 1.2. External Resources Supporting AI Literacy Development

External resources constitute a critical foundation for fostering AI literacy, enabling educational institutions to cultivate student proficiency by facilitating access to support. These resources encompass AI literacy courses, library resources, learning tools and platforms (Korte et al., 2024; Markus et al., 2024; Yim & Su, 2025). Comprehensive curricula or workshops that integrate technical competencies, ethical considerations, and societal implications of AI encourage responsible and meaningful student engagement (Korte et al., 2024; Markus et al., 2024). In addition, access to technological tools, advanced learning materials, and well-equipped classrooms are essential for implementing experiential and hands-on approaches to AI education (Yim & Su, 2025). Supportive environments foster creativity and innovation, positively influencing students’ engagement with AI content. Collectively, these resources create the structural and pedagogical conditions that enable students to acquire AI-related knowledge, skills, and ethical awareness.

Meanwhile, teachers play a central role in promoting AI literacy, as their knowledge, attitudes, and instructional support directly influence student learning outcomes. Research emphasizes that teachers must possess a thorough understanding of AI educational tools to facilitate effective instruction (Yim & Wegerif, 2024). Furthermore, teacher guidance, scaffolding, and thoughtful curriculum integration reduce challenges associated with AI adoption while enhancing student engagement and motivation (Liu et al., 2025). As demonstrated by Shen and Cui (2024), both technical and teacher support serve as critical catalysts for AI literacy in higher education by fostering students’ perceived autonomy and competence. In sum, resources provided by institutions and teachers provide access to knowledge, establishing the foundation for students to navigate and utilize AI competently and responsibly.

Taken together, the literature establishes that institutional resources and teacher support are associated with AI literacy, and on this basis we hypothesize that perceived resources and perceived teacher support predict students’ AI literacy (H1). It does not, however, establish whether these associations remain significant after adjustment for learners’ prior digital competence or psychological dispositions.

### 1.3. Digital Competence and AI Literacy

Digital competence, or digital literacy, constitutes the foundational skill set upon which AI literacy is built, providing the necessary groundwork for effective engagement with AI technologies. It is defined as consisting of the “skills and practices required to use new technologies in a meaningful way and as a tool for learning, working and leisure time, understanding the essential phenomena of digital technologies in society as well as in one’s own life, and the motivation to participate in the digital world as an active and responsible actor.” (Ilomäki et al., 2016, pp. 670–671). Beyond these basics, digital competence encompasses critical thinking, information management, collaboration, and problem-solving skills. AI literacy, in turn, is built upon digital and computer literacy frameworks, emerging as a distinct competency essential for navigating AI systems effectively and responsibly (Long & Magerko, 2020; Riina et al., 2022). The transition from digital literacy to AI literacy involves an evolution from general technological proficiency to advanced engagement with AI, requiring skills such as understanding algorithmic processes, interpreting AI outputs, and making informed decisions based on AI recommendations (Chee et al., 2025). This perspective conceptualizes AI literacy as a continuum rooted in digital competence, where each successive layer of skill development reinforces the capacity for responsible and effective AI use.

While empirical research has not yet been conducted extensively among university students, converging evidence from adjacent populations supports this proposition, and it is worth setting that evidence out in some detail. In a survey of university teachers, Dringó-Horváth et al. (2025) found digital competence to be a substantial predictor of AI literacy, with the strength of the association moderated by gender, age, professional experience, and discipline. Hava and Babayiğit (2024) similarly reported that teachers’ general digital proficiency predicted their competence in AI-related technological pedagogical content knowledge, indicating that AI-specific capability is built upon, rather than developed independently of, prior digital skill. Among researchers, Seker et al. (2025) found that digital literacy and data literacy jointly mediated the relationship between twenty-first century skills and AI literacy, which suggests that general digital capability functions as a pathway to AI competence rather than merely co-occurring with it. Evidence from student populations remains sparser. Hu et al. (2025) revealed that AI literacy is associated with computational thinking with a small but significant effect size in a sample of Chinese university students, and Celik (2023) found that computational thinking, cognitive absorption, and digital divide indicators each predicted AI literacy, concluding that it is “essential to analyse the factors underlying AI literacy from multiple perspectives” (p. 2). Taken together, these findings support the assumption that digital competence forms the essential foundation for AI literacy, which subsequently emerges as a higher-order and distinct skill set, and we accordingly hypothesise that digital competence predicts AI literacy after external resources are controlled (H2). This literature nonetheless shares the limitation identified above: none of these studies models psychological dispositions concurrently, so it remains unknown whether the contribution of digital competence is independent of the traits, emotions, and beliefs with which it is correlated.

### 1.4. Psychological Profile Related to AI Literacy

Besides external resources and prior digital competence, significant research efforts have been dedicated to investigating how individual psychological factors affect AI literacy. For instance, researchers have examined the role of mindfulness (Lan et al., 2025) and emotional intelligence (Fu, 2025). Lan et al. (2025) argued that mindfulness, defined as the “non-judgmental awareness of present experiences”, contributes to AI literacy by enabling individuals to engage more deeply with information, thereby fostering an autonomous awareness of intelligent technologies (p. 9). Similarly, Fu (2025) found that emotional intelligence, defined as the capacity to recognize, understand, and manage one’s own emotions while empathizing with others, facilitates AI literacy. Taken together, recent research showed that AI literacy is a skill that is subject to the disposition of how individuals process information.

These psychological profiles, including personal traits, emotional responses, and cognitive beliefs, shape how individuals perceive, engage with, and benefit from AI technologies, affecting both knowledge acquisition and the application of AI tools in educational and professional contexts. However, limited psychological research has integrated the external factors necessary to achieve a multi-perspective view of AI literacy, as advocated by Celik (2023). Furthermore, recent psychological frameworks suggest that rather than adhering to a single theoretical lens, research on individual differences could adopt a more integrative approach. By combining traits, motives, emotions, cognition, self-control, and contextual factors, researchers can better reflect the adaptive and comprehensive nature of the human experience (Mayer, 2020; Lukaszewski et al., 2020). Among these psychological factors, we propose that three, personal innovativeness, technology anxiety, and growth mindset in technology, have emerged as particularly salient in predicting engagement with and competence in AI.

Personal innovativeness, in a technological context, is a stable trait that reflects an individual’s propensity to embrace new ideas and adopt emerging technologies (Aldahdouh et al., 2020). Personal innovativeness serves as a key individual enabler within technology-acceptance models, shaping attitudes, perceived control, and intentions to utilize AI tools (Ma & Lei, 2024; C. Wang et al., 2024). Furthermore, by reducing fear of innovation, it also facilitates the adaptability required for university students to cultivate comprehensive AI literacy (Polat, 2025). By fostering a willingness to experiment and reducing resistance to change, personal innovativeness serves as a key psychological driver of AI literacy, although its influence may vary depending on the complexity of AI systems and contextual factors.

Technology anxiety encompasses feelings of apprehension, fear, or stress when interacting with new technologies, including AI (Wilson et al., 2023). Although direct empirical links between technology anxiety and AI literacy remain unexplored, this negative emotion is frequently associated with unfavorable attitudes and diminished usage (Ayed et al., 2025). Conversely, recent investigations into AI-specific anxiety, encompassing concerns over learning, configuration, job displacement, and sociotechnical blindness, suggest a paradoxical positive effect on motivated learning (Chen et al., 2024). These diverging findings point to the need for further empirical investigation to clarify how different dimensions of anxiety uniquely influence the development of AI literacy. Two considerations lead us to expect a more differentiated relationship than a simple negative association. First, generative AI systems are operated through natural language and require no programming expertise (Laupichler et al., 2022), so the operational barrier that technology anxiety is ordinarily held to raise is comparatively low in this domain. Second, anxiety is characterised in affective science as a vigilance state that biases attention towards potential threat and increases the processing of threat-relevant information (Eysenck et al., 2007). There is no obvious reason why such vigilance should impair the evaluative and ethical facets of AI literacy, and some reason to expect that it might sharpen them, since the critical appraisal of AI outputs and the recognition of ethical risk both depend on attending to what might go wrong. We therefore anticipate that technology anxiety may relate differently to the evaluative and to the performance-oriented dimensions of AI literacy, and we examine this possibility directly in our subdimension analyses.

Growth mindset in technology, or digital mindset, which reflects individuals’ beliefs about the malleability of their technological abilities (Solberg et al., 2020; Pybus & Gillan, 2015), might also shape engagement with AI systems. Learners who adopt a growth mindset, believing that technological skills can be developed through effort, tend to approach challenges in the process of learning new technologies as opportunities for learning and experimentation (Chow & To, 2025; Dinh, 2024). Conversely, a fixed mindset may lead to avoidance behaviours and apprehension, limiting the acquisition of AI skills. While there is no direct evidence linking growth mindset in technology to AI literacy, empirical studies show that interventions targeting growth mindset positively influence technology use (Dang & Liu, 2022).

In summary, we propose that psychological factors such as personal innovativeness, technology anxiety, and growth mindset in technology play a central role in shaping AI literacy. Innovativeness fosters proactive engagement and adoption, anxiety creates barriers to learning and exploration, and implicit beliefs influence motivation and resilience in interacting with AI systems. The current study examines the predictive value of these psychological factors above and beyond external resources and prior digital competence. On this basis, we hypothesise that personal innovativeness, technology anxiety, and growth mindset in technology predict AI literacy after external resources and prior digital competence are controlled (H3). As with the two preceding studies, the studies reviewed here share the limitation of examining psychological determinants without concurrent measurement of the external and experiential factors with which they are correlated, and the present study is designed to remedy this.

### 1.5. An Integrative Pedagogical Framework for AI Literacy Development

The central proposition of Social Cognitive Theory (Bandura, 1986) is triadic reciprocal determinism, which holds that human functioning is shaped by the continuous interaction of environmental determinants; personal determinants comprising cognitive, affective, and dispositional factors; and enactive behavioural experience. The three blocks of proposed determinants map directly onto the components of this framework. Perceived resources and perceived teacher support represent the environmental determinants. In pedagogical terms these constitute the learner-perceived instructional environment: institutional provision establishes the material conditions for engagement with AI, while teacher support functions as instructional scaffolding, guiding learners through tasks they could not yet undertake independently and supporting their sense of autonomy and competence in doing so (Liu et al., 2025; Shen & Cui, 2024). Prior digital competence represents enactive mastery experience, which Bandura identifies as the most influential source of perceived capability, since successful prior performance in a related domain supplies direct evidence of what one is able to accomplish. Personal innovativeness, technology anxiety, and growth mindset in technology represent, respectively, the dispositional, affective, and cognitive facets of personal determinants. Celik’s (2023) multidimensional account of AI literacy, on which the present study builds, is itself situated within this tradition; making that grounding explicit clarifies why these particular domains belong together in a single model.

**Figure 1:**
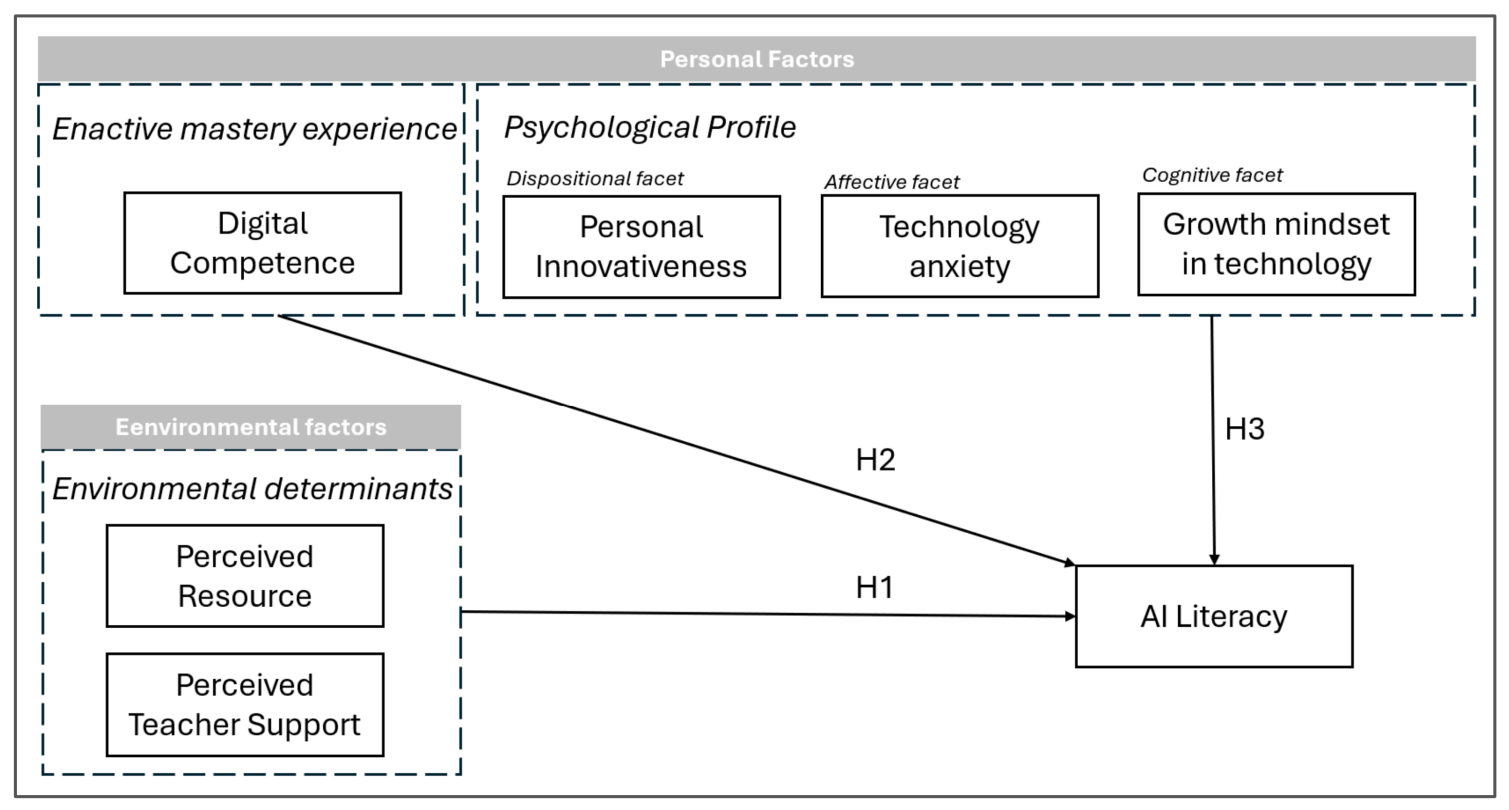
Conceptual Model of our Study.

This framework can additionally be read through the multi-level digital divide perspective, which distinguishes inequalities of resources from inequalities of skill and, in turn, from inequalities in the outcomes that resources and skill make possible (Van Dijk, 2020; Scheerder et al., 2017). Our three groups of determinants correspond closely to that framework, and the correspondence connects our findings to questions of equity in AI education. If learner-level determinants carry appreciable weight, then equalising access alone will not equalise outcomes. Figure 1 presents the resulting conceptual model.

Despite growing research on AI literacy, most studies have examined external resources, digital competence, and psychological factors in isolation, and certain psychological determinants, such as personal innovativeness and growth mindset in technology, remain understudied despite their potential to provide valuable insights into the development of AI literacy. To address these gaps, the present study adopts an integrative approach, examining how external resources, prior digital competence, and psychological profiles collectively shape AI literacy among undergraduates in a liberal arts university. By investigating these factors simultaneously, the study aims to provide a more comprehensive understanding of the determinants of AI literacy, offering empirical evidence to guide targeted interventions and curriculum design that promote responsible and effective engagement with AI technologies. The hypotheses of this study are as follows:

**H1.**  *Perceived resources and perceived teacher support significantly predict students’ AI literacy.*

**H2.**  *Digital competence significantly predicts students’ AI literacy after controlling for perceived resources and perceived teacher support.*

**H3.**  *Personal innovativeness, technology anxiety, and growth mindset in technology significantly predict AI literacy after controlling for external resources and prior digital competence.*

## 2. Materials and Methods

### 2.1. Participants and Procedures

Data were collected via an online survey administered through Qualtrics from December 2024 to January 2025. Undergraduates at a Hong Kong liberal arts university were invited to participate via email, which included a survey link and informed consent information. At the time of data collection, in December 2024 and January 2025, the institution’s approach to generative AI was as follows: The university where this research was conducted actively encourages the integration of generative AI in teaching and assessment to enhance student learning while safeguarding academic integrity. Students were provided with an AI subscription that they can use in courses, while faculty are encouraged to adapt assessment designs that promote critical AI literacy and effective, responsible technology application. On the other hand, the institution required faculty to clearly define permitted AI usage in course assignment guidelines, and students needed to submit a formal declaration of GenAI use. These arrangements were situated within a broader Hong Kong higher education environment in which institutions had, by late 2024, generally moved from initial restriction towards regulated integration of generative AI in learning and assessment. Data was collected approximately two years after the public release of ChatGPT, by which point most students had encountered generative AI tools.

The survey took approximately 20 min to complete, and participation was voluntary. Participants who completed the survey received a USD 7 incentive. The study received ethical approval from the university’s institutional ethics board (Human Research Ethics Committee (HREC) of Hong Kong Shue Yan University. Reference number: HREC 24-3(2), approval date 25 April 2025). All data were anonymized, and participants could withdraw at any time without penalty. A total of 303 undergraduate students participated. Inclusion criteria required completion of all survey items, and all responses met this requirement, resulting in no exclusions. Participant demographics are summarized in Table 1. The disciplinary composition of the sample was markedly uneven. Students enrolled in the social sciences (46.2%) and the arts (28.4%) together accounted for 74.6% of participants, whereas science, engineering, and medicine together accounted for fewer than 3%. This distribution reflects both the disciplinary profile of the liberal arts institution at which the study was conducted and the self-selection inherent in voluntary participation.

### 2.2. Measures

The dependent variable in this study is AI literacy. Furthermore, six independent variables were grouped into three categories: External Resources, Digital Competence, and Psychological Characteristics. All variables were assessed using validated scales originally developed for English-speaking populations, with minor adaptations for the AI context. Scales were translated into Traditional Chinese by the two authors through two rounds of forward and backward translation to ensure linguistic accuracy. Composite scores for each variable were calculated by averaging item responses. All scales demonstrated satisfactory internal reliability (α > 0.70).

#### 2.2.1. AI Literacy

AI literacy was measured using an adapted version of the ChatGPT Literacy Scale (Lee & Park, 2024). This scale frames AI literacy as a critical skill for communication and education, comprising 25 items across five subscales including technical proficiency, critical evaluation, communication proficiency, creative application, and ethical competence. The scale was developed through focus group discussions, expert reviews, exploratory factor analysis (EFA), and confirmatory factor analysis (CFA). A sample item from the scale is “I can train and fine-tune Generative AI tools for specific purposes or applications.” Items were rated on a 5-point Likert scale (1 = Strongly Disagree, 5 = Strongly Agree). For this study, we focused on overall AI literacy by averaging all items.

#### 2.2.2. External Resources

Perceived Resources: Measured with 4 items adapted from Sivo et al. (2018), assessing the availability of digital resources in university courses. A sample item from the scale is “I have the resources I would need to use digital tools in the courses.” Items were rated on a 7-point Likert scale (1 = Extremely Unlikely, 7 = Extremely Likely).

Perceived Teacher Support: Measured with 3 self-designed items evaluating teacher support for using digital tools in academic settings. A sample item from the scale is “Teachers in my university encourage me to use digital tools to learn in the courses.” Items were rated on a 5-point Likert scale (1 = Strongly Disagree, 5 = Strongly Agree).

#### 2.2.3. Digital Competence

Digital Competence was assessed using 28 items from the Students’ Digital Competence Scale (SDiCoS; Tzafilkou et al., 2023). The original scale includes five subscales including information and data literacy, Communication and Collaboration, Digital Content Creation, Safety, and problem solving. A sample item from the scale is “I can evaluate an object and/or a smart device using appropriate quality criteria (e.g., authenticity, utility, easy to use, appearance, functionality, enjoyment).” Items were rated on a 5-point Likert scale (1 = Strongly Disagree, 5 = Strongly Agree). For analyses, we used an overall digital competence score by averaging all items.

#### 2.2.4. Psychological Characteristics

In our study, personal innovativeness, technology anxiety and growth mindset in technology were assessed. Personal Innovativeness was measured with 4 items from Xie (2022), assessing openness to new technologies. A sample item from the scale is “I am curious about many things.”. Items were rated on a 5-point Likert scale (1 = Strongly Disagree, 5 = Strongly Agree).

Technology Anxiety was measured with 11 items from Wilson et al. (2023), evaluating discomfort with technology use. A sample item from the scale is “I am reluctant to learn new features of technology.” Items were rated on a 5-point Likert scale (1 = Strongly Disagree, 5 = Strongly Agree). Higher scores indicate greater anxiety.

Growth Mindset in Technology was measured with 5 items from Pybus and Gillan (2015), assessing beliefs about learning ability in technology. A sample item from the scale is “Practice, hard work, effort, and persistence can change your ability to use technology.” Items were rated on a 7-point Likert scale (1 = Strongly Disagree, 7 = Strongly Agree). This reflects a growth mindset in technology.

### 2.3. Data Analysis

We conducted hierarchical multiple regression analyses using SPSS 28 to examine the effects of external support, digital competence, and psychological characteristics on overall AI literacy. The analysis was structured in four steps to test the incremental contribution of each category of predictors.

In the first step, gender and grade level were entered into the model. In the second step, external support variables, including perceived resources and perceived teacher support, were entered into the model. In the third step, digital competence was added to assess its additional explanatory power beyond external support. In the fourth step, psychological characteristics, comprising personal innovativeness, technology anxiety, and growth mindset in technology, were included to evaluate their incremental contribution to predicting AI literacy.

Hierarchical regression was chosen to allow a systematic assessment of how each block of variables contributes to explaining variance in AI literacy. Model fit was evaluated using *R*^2^, adjusted *R*^2^, F-statistics, and *p*-values, while changes in *R*^2^ were examined to determine the additional variance accounted for at each step. Standardized beta coefficients, standard errors, t-values, and *p*-values were calculated for all predictors to assess their relative contributions and statistical significance.

## 3. Results

The results are reported in three stages. First, we present descriptive statistics, internal consistency estimates, and zero-order correlations, which establish the bivariate relationships against which the multivariate findings should be read. Second, we report the hierarchical regression predicting total AI literacy, testing H1, H2, and H3 in sequence, with each block of predictors entered in the order specified by the framework set out in Section 1.5. Third, we report parallel analyses for each of the five subdimensions of the AI literacy measure; these are presented last because they qualify and refine the composite findings rather than replacing them.

### 3.1. Preliminary Analysis

The results of descriptive statistics and correlations are presented in Table 2. All variables used in this study were significantly correlated (*p* < 0.05). Specifically, AI literacy was positively correlated with perceived resources, perceived teacher support, digital competence, personal innovativeness, and growth mindset in technology while being negatively correlated with technology anxiety.

### 3.2. Hierarchical Multiple Regression Analysis

As displayed in Table 3, the hierarchical regression analysis involved four models. Model 1 comprised the covariates of gender and grade level. Model 2 added perceived resources and perceived teacher support, providing the test of H1. Model 3 added digital competence, providing the test of H2. Model 4 added the psychological profile, comprising personal innovativeness (trait), technology anxiety (emotion), and growth mindset in technology (belief), providing the test of H3. The dependent variable in each model was total AI literacy.

In model 1, the F test showed that the model of gender and grade level was not statistically significant for predicting AI literacy among participants (*F* = 2.00, *p* > 0.05). R-squared in model 1 was 0.01.

In model 2, the F test showed that perceived resources and perceived teacher support significantly predicted AI literacy among participants (*F* = 30.44, *p* < 0.001). The model shows significant improvement over the first model ∆*F* (2, 298) = 58.13, *p* < 0.001. The R-square value of model 2 is 0.29 in contrast with 0.01 in model 1, suggesting that gender, grade level, perceived resources, and perceived teacher support account for 29% of variance. Both perceived resources and perceived teacher support demonstrated significant positive predictive effect on AI literacy. H1 was therefore supported. Both perceived resources and perceived teacher support significantly predicted AI literacy when entered after the covariates.

In model 3, F test (*F* = 28.52, *p* < 0.001) showed that the digital competence and external resources variables also significantly predicted AI literacy among students in higher education. The model shows significant improvement over the second model ∆*F* (1, 297) = 15.10, *p* < 0.001. The R-square value of model 3 is 0.32 in contrast with 0.29 in model 2. H2 was therefore supported. Digital competence significantly predicted AI literacy after perceived resources and perceived teacher support were controlled. It is notable that the coefficient for perceived resources fell from 0.39 to 0.24 upon its entry while remaining significant.

In model 4, F test (*F* = 23.72, *p* < 0.001) showed that the psychological variables added also significantly predicted AI literacy among students in higher education. The model shows significant improvement over the third model ∆*F* (3, 294) = 10.93, *p* < 0.001. The R-square value of model 4 is 0.39 in contrast with 0.32 in model 3. Tests to see if the data met the assumption of collinearity indicated that multicollinearity was not a concern (Gender, Tolerance = 0.97, VIF = 1.03; Grade Level, Tolerance = 0.96, VIF = 1.04; Perceived resources, Tolerance = 0.44, VIF = 2.27; Perceived Teacher Support, Tolerance = 0.71, VIF = 1.41; Digital Competence, Tolerance = 0.48, VIF = 2.08; Personal Innovativeness, Tolerance = 0.65, VIF = 1.53; Technology Anxiety, Tolerance = 0.70, VIF = 1.44; Growth Mindset in Technology, Tolerance = 0.59, VIF = 1.71). H3 was therefore supported. Personal innovativeness, technology anxiety, and growth mindset in technology each significantly predicted AI literacy after external resources and prior digital competence were controlled. Both external predictors remained significant in the final model, though attenuated relative to Model 2 (perceived resources β = 0.15; perceived teacher support β = 0.15). Technology anxiety emerged as a positive predictor (β = 0.15, *p* < 0.01) notwithstanding its negative zero-order correlation with AI literacy (r = −0.14, *p* < 0.05).

### 3.3. Subdimensional Analysis

Because AI literacy is conceptualised as a multidimensional construct, the four-step model was refitted separately for each of the five subdimensions of the AI Literacy Scale. Final-model coefficients are reported in Table 4. All five models were significant, with explained variance ranging from 0.26 to 0.32. Specifically, the combination of external conditions, digital competence, and psychological profile significantly predicted Technical Proficiency (*R*^2^ = 0.24, *F* = 12.63, *p* < 0.001), Critical Evaluation (*R*^2^ = 0.28, *F* = 14.04, *p* < 0.001), Communication Proficiency (*R*^2^ = 0.31, *F* = 16.51, *p* < 0.001), Creative Application (*R*^2^ = 0.31, *F* = 16.17, *p* < 0.001), and Ethical Competence (*R*^2^ = 0.32, *F* = 17.42, *p* < 0.001).

The psychological block contributed significantly to all five subdimensions, which confirms that its explanatory value is not an artefact of aggregation. Individual predictors, however, operated selectively. Personal innovativeness significantly predicted technical proficiency, critical evaluation, communication proficiency, and ethical competence, most strongly technical proficiency (β = 0.26, *p* < 0.001).

Growth mindset in technology predicted four of the five subdimensions, most strongly ethical competence (β = 0.27, *p* < 0.001). Digital competence predicted critical evaluation, creative application, and ethical competence, but not technical proficiency or communication proficiency. Perceived resources predicted only communication proficiency (β = 0.22, *p* < 0.001) and creative application (β = 0.17, *p* < 0.05). All three psychological variables predicted ethical competence.

The pattern for technology anxiety is of particular interest. Its positive association was confined to critical evaluation (β = 0.14, *p* < 0.05) and ethical competence (β = 0.20, *p* < 0.001) and was non-significant for technical proficiency, communication proficiency, and creative application. The positive residual effect observed in the composite model thus arises entirely from the two evaluative dimensions and is absent from the three performance-oriented dimensions.

## 4. Discussion

The study investigated how AI literacy is affected by environmental factors, prior knowledge, and psychological profiles. With generative AI becoming more common in professional and daily life, understanding how AI literacy can be cultivated and the underlying factors for individual differences is crucial. While previous scholarship has often examined the influence of external resources, prior digital competence, and psychological factors in isolation, this study provides a novel integration of these variables. By analysing them concurrently, this article elucidates their distinctive effects on AI literacy, filling a critical gap in the existing literature.

### 4.1. Multifaceted Determinants of AI Literacy: Insights from Hierarchical Regression

Our results indicate that AI literacy is a product of contextual, cognitive and psychological factors. Specifically, the hierarchical regression analysis revealed that external factors, including teacher support and perceived resources, initially contribute to AI literacy, but their influence diminishes subsequently when accounting for students’ digital competencies and psychological profiles. Our findings reveal that while external factors provide initial scaffolding for AI literacy, digital competence further enables effective utilization of AI systems based on their experience. This is also evident in previous research showing how computational thinking and 21st-century skills contribute to AI literacy (Celik, 2023; Hu et al., 2025; Seker et al., 2025), suggesting that prior digital exposure not only bridges gaps in technical proficiency but also amplifies the benefits of environmental supports. Psychological profiles further refine this process (Chen et al., 2024). The result can contribute to the existing body of literature investigating the factors affecting AI literacy.

First, these findings support the suggestion made by Celik (2023) that AI literacy is a multifaceted phenomenon, noting that it is “essential to analyse the factors underlying AI literacy from multiple perspectives” (p. 2). While extensive research has been dedicated to exploring the determinants of AI literacy including external factors (Dringó-Horváth et al., 2025; Hava & Babayiğit, 2024; Seker et al., 2025) and psychological variables (Fu, 2025; Lan et al., 2025; K. Wang et al., 2025), few studies have integrated these perspectives to investigate how AI literacy emerges from the interplay between external resources and an individual’s psychological state (Celik, 2023; Shen & Cui, 2024). Our research contributes to this discourse by demonstrating the synergistic effects of external resources, prior experience, and psychological profiles.

Second, the effects of external support and digital competence remained statistically significant even after controlling for personal innovativeness, technology anxiety, and growth mindset of technology. These results underscore the epistemic foundations of AI literacy (Long & Magerko, 2020); while dispositional psychological traits serve as potent drivers of engagement, they do not supersede the fundamental necessity of foundational knowledge or the social construction of expertise. This suggests that psychological readiness is a complement to, rather than a substitute for, objective technical competency and environmental support.

Third, these results broaden the empirical evidence regarding the impact of digital competence on AI literacy. While previous research has established that digital competence facilitates the development of AI literacy among teachers and researchers (Dringó-Horváth et al., 2025; Hava & Babayiğit, 2024; Seker et al., 2025), our findings extend it to undergraduate students, and do so under the more demanding condition of simultaneous adjustment for environmental and psychological determinants. Furthermore, digital competence predicted critical evaluation, creative application, and ethical competence, but not technical proficiency or communication proficiency. These findings challenge any straightforward reading of digital competence as a general technical foundation, and suggest instead that its contribution operates through judgement and application rather than through operational facility with the tools themselves. Nevertheless, we are not in a position to claim that the role of digital competence is a universal prerequisite consistent across educational roles and academic levels, as a single-institution sample drawn predominantly from two disciplinary areas cannot establish universality.

### 4.2. The Role of Innovativeness, Anxiety, and Mindset in Mastering AI for University Students

On the other hand, our results highlight the critical influence of psychological profiles on AI literacy, particularly through personal innovativeness, technology anxiety, and growth mindset in technology. While personal innovativeness, technology anxiety, and growth mindset have strong theoretical impacts on AI literacy, the empirical associations remain underexplored. This study addresses this gap by providing empirical evidence of how technology anxiety and growth mindset in technology uniquely contribute to the development of AI literacy. Taken together, these elements exemplify how cognitive, emotional, and personality factors are essential in developing expertise in AI.

First, personal innovativeness predicted AI literacy, which is consistent with research using technology acceptance frameworks such as TAM and UTAUT II (Ma & Lei, 2024; C. Wang et al., 2024). That work concerns the intention to adopt AI tools rather than competence in using them, so our result extends it rather than simply confirming it: the disposition to try new technology is associated not only with taking AI up but with knowing what to do with it. The trait appears to operate through use: innovative students experiment more, and operational skill accumulates from that experimentation. This is consistent with the conceptualisation of innovativeness as a propensity for hands-on engagement with unfamiliar technology (Aldahdouh et al., 2020), and it suggests that the benefit is largely confined to what practice can teach. It also identifies where the risk of falling behind lies, namely with students who are capable but cautious, and for whom exposure will not occur unprompted. The cross-sectional design cannot rule out the reverse ordering, in which greater technical facility leads students to describe themselves as innovative.

Second, and contrary to the direction that the technology anxiety literature would lead one to expect, technology anxiety was a positive predictor of AI literacy in the full model. In our subdimensional analysis, the positive association was confined to critical evaluation and ethical competence and was absent from technical proficiency, communication proficiency, and creative application. Anxious students, in other words, were not better at operating generative AI; they were more attentive to what it might get wrong. This pattern is consistent with accounts of anxiety as a vigilance state that biases attention towards potential threat and increases the processing of threat-relevant information (Eysenck et al., 2007), and with evidence that AI-specific anxiety can motivate rather than inhibit engaged learning (Chen et al., 2024).

Third, our findings indicate that growth mindset in technology exerts a significant positive influence on AI literacy. While the role of a growth mindset in technological contexts has historically received less attention, it is increasingly recognized as a critical cognitive driver of AI adoption (Solberg et al., 2020; Pybus & Gillan, 2015). Previous research suggests that individuals possessing a growth mindset believe that sustained effort facilitates the mastery of digital tools, which in turn encourages experimentation and learning-oriented behaviours (Chow & To, 2025; Dinh, 2024). This study extends this line of inquiry by demonstrating that a growth mindset positively predicts AI literacy. Specifically, students who perceive AI competencies as developable are not only more inclined to engage with the technology but also more likely to achieve proficiency. Given that these beliefs are malleable and can be cultivated through intervention (Dang & Liu, 2022), our results suggest that fostering such mindset may be a vital strategy for unlocking AI potential across diverse learner populations, regardless of their initial resources or prior digital experience.

### 4.3. Practical Implications

Current reviews indicate that the dominant institutional strategy for promoting AI literacy remains centred on formal curricula and coursework (S. Zhang et al., 2025). Such initiatives tend to prioritise the question of what to teach over the questions of how to teach it and whom to teach it to. Our findings speak principally to the latter two.

The first implication concerns the limits of provision. Perceived resources and perceived teacher support together explained 28% of the variance in AI literacy, and both remained significant in the full model, so institutional provision plainly matters. The subdimension analyses nonetheless suggest that its reach is narrower than is often assumed: perceived resources predicted only communication proficiency and creative application, and perceived teacher support predicted only communication proficiency and ethical competence. Investment in infrastructure and access should therefore be understood as addressing particular components of AI literacy rather than as a general remedy, and should be accompanied by measures directed at learners themselves.

The second implication concerns beliefs about ability. Growth mindset in technology predicted four of the five subdimensions and was the most consistent psychological predictor in the study. Since such beliefs are malleable and can be shifted through intervention (Dang & Liu, 2022), and since messages framing technological competence as developable through effort can be embedded in existing curricula at low cost, this represents the intervention with the broadest reach relative to the effort required. Chow and To (2025) also suggested that a technological growth mindset is associated with generative AI use in higher education, and our findings extend this to competence rather than usage.

The third implication concerns anxiety, and is the least intuitive. The prevailing assumption is that technology anxiety should be reduced. Our results complicate this. Once other determinants were controlled, the residual component of anxiety was positively associated with precisely those dimensions of AI literacy concerned with critical appraisal and ethical judgement. Undifferentiated efforts to make students comfortable with AI may therefore carry a cost if what is removed along with apprehension is the disposition to scrutinise. The more defensible aim is to address anxiety that is debilitating, through supportive and low-stakes introductory experiences (Elsayed et al., 2024), while preserving the critical stance that appears to accompany it. Framing caution as a component of competent AI use, rather than as an obstacle to it, may be the more productive pedagogical posture.

Nevertheless, it is important to note that the above practical implications are generated by cross-sectional correlational data, and none of them has been tested experimentally. Establishing whether interventions of the kinds described actually raise AI literacy, and for which students, requires designs capable of supporting causal inference. We offer them as a more specific starting point than the general exhortation to adopt a holistic approach.

### 4.4. Limitations and Future Directions

However, there are several limitations that should be noted in our study. First, the study is correlational in nature. The sample for our study was obtained through a cross-sectional design. Therefore, causal relationships cannot be confirmed in our study. This limitation aligns with broader concerns in AI literacy research, where cross-sectional approaches, while useful for identifying associations, fail to establish directionality or temporal precedence among factors such as external conditions, digital competence, and psychological profiles (Celik, 2023). Additionally, this design does not account for how cultural factors specific to Hong Kong, such as high technology adoption rates or educational pressures, might influence results, potentially limiting generalizability to other regions. Future studies could employ longitudinal designs to track changes in AI literacy over time, examining how factors like teacher support and personal innovativeness evolve and influence literacy development, as suggested in explorations of self-efficacy and engagement (Chen et al., 2024). Experimental interventions, such as randomized training programs targeting growth mindset, could also test causal effects on AI literacy subscales.

Second, the study used a non-randomized sampling method. In our study, participants joined the research through invitations via email and posters on campus at a single liberal arts university. While this convenience sampling approach allowed for efficient data collection from university students in Hong Kong, it may introduce selection bias, potentially overrepresenting individuals with a higher interest in or exposure to AI technologies, thereby limiting the representativeness of the sample. Furthermore, because data collection was restricted to one specific institution, the unique student background and institutional culture may further limit the generalizability of the findings. Individual differences in AI literacy, shaped by demographics such as nationality and prior experience, further highlight the need for caution in generalizing findings beyond this context (Almatrafi et al., 2024). To address this, future research should incorporate stratified or random sampling techniques across diverse populations, including varying disciplines and socioeconomic backgrounds, to better capture disparities in AI competency. Importantly, social sciences and arts students together comprised 74.6% of participants. Disciplinary background is plausibly associated with both prior digital competence and AI literacy, so our findings should be understood as characterising AI literacy development among students in the humanities and social sciences rather than among undergraduates generally. This composition also restricts the range on several study variables, and the associations we report are therefore likely to be conservative rather than inflated. The predominance of female participants (70.6%) is a further constraint on generalisability, though gender did not significantly predict AI literacy in any model. Meanwhile, students in the humanities and social sciences are comparatively underrepresented in the AI literacy literature, which has concentrated on computing, engineering, and other STEM populations. Since these students will increasingly encounter generative AI, and typically do so with less formal technical preparation, their AI literacy development is a substantive question in its own right. We nonetheless treat this composition as defining the scope of our claims rather than as a feature of the design. Future research should employ discipline-stratified sampling across multiple institutions to establish whether the relative contributions of external resources, digital competence, and psychological profile vary by field of study.

Third, the AI literacy assessment tools used in this study require further validation in the Chinese context. The instrument employed in our study was developed by Lee and Park (2024) as an English-language measure originally validated in Korean society. Although emerging efforts have sought to conceptualise and operationalise AI literacy, no scales have yet been developed within Chinese society (Lintner, 2024). Measurement invariance between the original English instrument and our Traditional Chinese version has likewise not been established, and, as noted in Section 2.3, no cognitive pretesting was undertaken to confirm that items were interpreted as intended. Future work in this setting should establish both the factor structure and its invariance before drawing comparisons across linguistic or cultural groups.

Fourth, all constructs were assessed by self-report within a single instrument at a single time point, which raises the possibility of common method variance. This is a particular consideration for our outcome, since the ChatGPT Literacy Scale measures self-perceived rather than demonstrated competence, and self-assessed competence may be influenced by the same dispositional factors that appear as predictors in our models. We did not include a marker variable or conduct a formal test of common method bias, and we cannot therefore quantify its contribution. Future studies would be strengthened by incorporating performance-based or behavioural measures of AI literacy alongside self-report. Furthermore, while the forward-and-backward translation procedures ensured the linguistic equivalence of the survey instruments, the adoption of originally English-language scales within the Hong Kong higher education context necessitates careful reflection on broader conceptual and cultural dynamics. Specifically, constructs such as personal innovativeness, technology anxiety, and growth mindset are embedded within Western psychological frameworks that may interact differently with local cultural values. In Hong Kong, high academic expectations, distinct attitudes toward institutional authority, and culturally specific perceptions of academic risk and face-saving can influence how students interpret and respond to self-reported questionnaire items. Consequently, while these validated instruments capture critical dimensions of AI literacy, their cross-cultural application must be interpreted with an awareness of the unique socio-educational environment that shapes university students’ technological experiences in the region.

Fifth, the social cognitive framework and our data capture the learner-perceived pedagogical environment rather than observed instructional practice. We also did not measure curriculum design, instructional approach, assessment design, or instructors’ own AI-related pedagogical competence, each of which is likely to shape AI literacy development and none of which can be inferred from students’ perceptions of resources and support. Direct measurement of these pedagogical variables, ideally through designs that link student outcomes to characteristics of the instruction they actually received, is a priority for future research and would permit a considerably stronger test of the framework advanced here.

## Figures and Tables

**Table 1:** Demographic of participants.

	Frequency	Percentage
Gender		
Male	89	29.4
Female	214	70.6
Grade Level		
Year 1	78	25.7
Year 2	83	27.4
Year 3	70	23.1
Year 4	70	23.1
Year 5 or above	2	0.7
Major of Study		
Social Sciences	140	46.2
Arts	86	28.4
Business Administration	61	20.1
Science	7	2.3
Law	5	1.7
Other	2	0.7
Engineering	1	0.3
Medicine	1	0.3

Note. *N* = 303.

**Table 2:** Correlation and descriptive statistics of variables.

Variable	1	2	3	4	5	6	*M*	*SD*	*α*
AI literacy							3.47	0.70	0.96
2. Perceived Resources	0.50 ***						5.18	1.19	0.91
3. Perceived Teacher support	0.41 ***	0.49 ***					3.80	0.80	0.87
4. Digital Competence	0.50 ***	0.68 ***	0.46 ***				3.91	0.57	0.93
5. Personal Innovativeness	0.48 ***	0.50 ***	0.37 ***	0.51 ***			3.81	0.74	0.81
6. Technology anxiety	−0.14 *	−0.34 ***	−0.16 **	−0.29 ***	−0.21 ***		2.46	0.80	0.93
7. Growth mindset in technology	0.40 ***	0.47 ***	0.25 ***	0.41 ***	0.39 ***	−0.54 ***	4.91	0.97	0.74

* *p* < 0.05, ** *p* < 0.01, *** *p* < 0.001.

**Table 3:** Regression Analysis of AI Literacy.

Predictor Variable	β			
	Model 1	Model 2	Model 3	Model 4
Gender	−0.12 * (−2.00)	−0.06 (−1.23)	−0.05 (−1.09)	−0.04 (−0.85)
Grade Level	−0.01 (−0.24)	0.00 (0.04)	0.00 (0.011)	0.01 (0.15)
Perceived Resources		0.39 *** (6.82)	0.24 *** (3.45)	0.15 * (2.17)
Perceived Teacher support		0.22 *** (3.85)	0.17 ** (3.07)	0.15 ** (2.71)
Digital Competence			0.26 *** (3.89)	0.18 ** (2.79)
Personal Innovativeness				0.19 *** (3.45)
Technology anxiety				0.15 ** (2.74)
Growth mindset in technology				0.22 *** (3.75)
*F*	2.00	30.44 ***	28.52 ***	23.72 ***
*R* ^2^	0.01	0.29	0.32	0.39
Adjusted *R*^2^	0.01	0.28	0.31	0.38
∆*R*^2^	0.01	0.28	0.03	0.06
∆*F*	2.00	58.13 ***	15.10 ***	10.93 ***
Standard Error of Estimate	0.70	0.60	0.58	0.56

*t*-values are shown in parentheses. * *p* < 0.05, ** *p* < 0.01, *** *p* < 0.001.

**Table 4:** Regression Analysis of Subdimensions of AI Literacy.

	β				
	Predictor Variable				
	Technical Proficiency	Critical Evaluation	Communication Proficiency	Creative Application	Ethical Competence
Gender	−0.08 (−1.54)	−0.05 (−0.95)	0.01 (0.23)	−0.03 (−0.60)	0.00 (0.06)
Grade Level	0.03 (0.60)	0.01 (0.10)	0.00 (0.08)	0.03 (0.63)	−0.06 (−1.12)
Perceived Resources	0.05 (0.61)	0.12 (1.57)	0.22 *** (3.01)	0.17 * (2.33)	0.10 (1.39)
Perceived Teacher support	0.14 * (2.35)	0.08 (1.38)	0.14 * (2.48)	0.11 (1.95)	0.15 * (2.60)
Digital Competence	0.12 (1.71)	0.19 ** (2.66)	0.11 (1.57)	0.17 * (2.37)	0.20 ** (2.83)
Personal Innovativeness	0.26 *** (4.15)	0.15 * (2.50)	0.13 * (2.12)	0.11 (1.87)	0.13 * (2.12)
Technology anxiety	0.10 (1.62)	0.14 * (2.39)	0.11 (1.85)	0.10 (1.71)	0.20 *** (3.48)
Growth mindset in technology	0.12 (1.76)	0.20 ** (3.12)	0.19 ** (2.92)	0.21 *** (3.33)	0.27 *** (4.27)
*F*	12.63 ***	14.04 ***	16.51 ***	16.17 ***	17.42 ***
*R* ^2^	0.26	0.28	0.31	0.31	0.32
Adjusted *R*^2^	0.24	0.26	0.29	0.29	0.30
Standard Error of Estimate	0.77	0.71	0.69	0.68	0.68

*t*-values are shown in parentheses. * *p* < 0.05, ** *p* < 0.01, *** *p* < 0.001.

## Data Availability

The original data presented in the study are openly available in OSF at “https://osf.io/am7k3/” (accessed on 20 May 2026).

## Abbreviations

The following abbreviations are used in this manuscript:

AI	Artificial Intelligence
